# The Effect of α ^+^-Thalassaemia on the Incidence of Malaria and Other Diseases in Children Living on the Coast of Kenya

**DOI:** 10.1371/journal.pmed.0030158

**Published:** 2006-04-18

**Authors:** Sammy Wambua, Tabitha W Mwangi, Moses Kortok, Sophie M Uyoga, Alex W Macharia, Jedidah K Mwacharo, David J Weatherall, Robert W Snow, Kevin Marsh, Thomas N Williams

**Affiliations:** 1Kenya Medical Research Institute/Wellcome Trust Programme, Centre for Geographic Medicine Research Coast, Kilifi District Hospital, Kilifi, Kenya; **2**Weatherall Institute of Molecular Medicine, John Radcliffe Hospital, The University of Oxford, Oxford, United Kingdom; **3**Nuffield Department of Medicine, John Radcliffe Hospital, The University of Oxford, Oxford, United Kingdom; **4**Department of Paediatrics, John Radcliffe Hospital, The University of Oxford, Oxford, United Kingdom; Imperial CollegeUnited Kingdom

## Abstract

**Background:**

The α-thalassaemias are the commonest genetic disorders of humans. It is generally believed that this high frequency reflects selection through a survival advantage against death from malaria; nevertheless, the epidemiological description of the relationships between α-thalassaemia, malaria, and other common causes of child mortality remains incomplete.

**Methods and Findings:**

We studied the α ^+^-thalassaemia-specific incidence of malaria and other common childhood diseases in two cohorts of children living on the coast of Kenya. We found no associations between α ^+^-thalassaemia and the prevalence of symptomless Plasmodium falciparum parasitaemia, the incidence of uncomplicated P. falciparum disease, or parasite densities during mild or severe malaria episodes. However, we found significant negative associations between α ^+^-thalassaemia and the incidence rates of severe malaria and severe anaemia (haemoglobin concentration < 50 g/l). The strongest associations were for severe malaria anaemia (> 10,000 P. falciparum parasites/μl) and severe nonmalaria anaemia; the incidence rate ratios and 95% confidence intervals (CIs) for α ^+^-thalassaemia heterozygotes and homozygotes combined compared to normal children were, for severe malaria anaemia, 0.33 (95% CI, 0.15,0.73; *p* = 0.006), and for severe nonmalaria anaemia, 0.26 (95% CI, 0.09,0.77; *p* = 0.015).

**Conclusions:**

Our observations suggest, first that selection for α ^+^-thalassaemia might be mediated by a specific effect against severe anaemia, an observation that may lead to fresh insights into the aetiology of this important condition. Second, although α ^+^-thalassaemia is strongly protective against severe and fatal malaria, its effects are not detectable at the level of any other malaria outcome; this result provides a cautionary example for studies aimed at testing malaria interventions or identifying new malaria-protective genes.

## Abstract 2

A study of children from the coast of Kenya suggests that selection for α ^+^-thalassemia in the population might be mediated by a specific effect against severe anemia.

## Introduction

Although malaria causes more than 200 million episodes of fever among young African children every year, fewer than 1% of these episodes are fatal [ 1, 2]. The reasons that some children die, whereas most survive are complex and poorly understood; however, one approach that may help to understand this process more fully is to study the effects of natural host malaria-resistance factors.

The α ^+^-thalassaemias are some of the best-recognised malaria-protective polymorphisms [ 3], having risen to such high frequencies in many populations (as high as 80%) that they are now considered the commonest monogenic disorders of humans [ 4]. Nevertheless, both the mechanisms of protection and their specificity for malaria remain unknown. We recently described the effect of α ^+^-thalassaemia on the prevalence, incidence, and density of P. falciparum infections in children living in a malaria-endemic area on the coast of Kenya, and showed that these effects may be attenuated when coinherited with the sickle cell trait [ 5]. In the current article we aimed to further describe the effects of α ^+^-thalassaemia on the subtypes of severe falciparum malaria and on the incidence of other childhood illnesses.

## Methods

### Mild Disease Cohort

The mild disease cohort study has been described in detail previously [ 5– 7]. Briefly, participants were recruited from an age-stratified population sample and monitored subsequently by active weekly surveillance for clinical events from September 1998 until August 2001. Intercurrent clinical events were monitored through a dedicated research outpatient clinic. Children born into study households were recruited at birth, and participants exited if informed consent was withdrawn, if they moved away for more than 2 months, or if they died. In addition, we conducted four cross-sectional surveys, in March, July, and October 2000 and in June 2001, to assess the prevalence of P. falciparum parasites. This analysis is limited to 301 children who were under 5 y old for more than 1 wk during the study period, and on whom full data were available on both α ^+^-thalassaemia genotype and haemoglobin (Hb) S phenotype.

### Birth Cohort

The design and conduct of the birth cohort study has also been described in detail previously [ 8– 10]. Briefly, between May 1992 and April 1995 we recruited all children born within a defined rural study area to the north of our research unit, a larger part of the same geographic area in which the mild disease cohort study was conducted, into a fixed birth cohort. We recorded all admissions to the paediatric wards at Kilifi District Hospital (the closest hospital facility to the study area) from participants of this cohort until December 1997. Although routine blood sampling was not a part of the original design, between May and October 2000 we succeeded in identifying 2,695 resident surviving members of this cohort and invited them to provide a blood sample for haematological typing. Full typing for both HbS and α ^+^-thalassaemia was completed on 2,104 children.

### Clinical Definitions

Throughout both studies, trained clinicians assessed all participants as they presented, and collected clinical data onto standard proformas. Although we recorded up to three diagnoses on each child, our current analysis is limited to primary or secondary diagnoses.

In the mild disease cohort study, we defined symptomless parasitaemia on the basis of a slide positive for P. falciparum malaria in the absence of fever or other symptoms of clinical illness. We used two definitions for clinical malaria. Definition 1 included fever (axillary temperature > 37.5 °C) in conjunction with a slide positive for blood stage asexual P. falciparum parasites at any density at all ages. Definition 2 was fever in conjunction with a positive slide at any density in children under 1 y old or at a density of 2,500 parasites/μl in children over 1 y old. Definition 2, which was derived by multiple logistic regression as described previously [ 6], accorded with a sensitivity and specificity for clinical malaria of greater than 85% in the population as a whole; however, because we have no data that allow us to confirm that these definitions remain appropriate for children with α ^+^-thalassaemia, we have reported our analyses based on both this definition and the more inclusive definition 1.

Malaria was considered the primary diagnosis on hospital admission if P. falciparum parasites were found in the peripheral blood and other likely causes for clinical presentation could be excluded. Based on reference [ 11] with some modifications, we used the following definitions to describe severe malaria: (1) cerebral malaria, which included coma (the inability to localize a painful stimulus, assessed more than 1 h after a seizure or after the administration of anticonvulsants, and following correction of hypoglycaemia) or prostration (the inability to breast feed or sit without assistance); (2) multiple seizures, which included two or more seizures within 24 h of admission; and (3) hyperparasitaemia, a parasite density at which over 20% of red cells were infected.

We used two different definitions for severe malaria anaemia (SMA): (1) definition 1, which accorded with that used by the WHO [ 11]—a haemoglobin under 50 g/l in association with a parasite density of more than 10,000/μl, and (2) definition 2—a haemoglobin under 50 g/l, in association with a parasitaemia of any density. We defined severe nonmalaria anaemia as haemoglobin below 5.0 g/dl in the presence of a negative malaria blood smear. Upper respiratory tract infection, lower respiratory tract infection, gastroenteritis, and helminth and skin infection were defined as described previously [ 12]. In the mild disease cohort study, slide-negative fever was defined as a temperature higher than 37.5 °C, in the presence of a negative malaria slide, in a participant who had not received treatment with an antimalarial drug within the preceding 21 d. This definition took no account of the primary or secondary diagnosis, and therefore encapsulated febrile episodes from a range of nonmalaria causes. Fever of unknown cause was a diagnosis of exclusion, allocated to children with a negative malaria slide and no obvious explanation for their fever.

### Laboratory Procedures

Blood smears were examined for malaria parasites using standard methods. Parasite densities were recorded as a ratio of parasites to white blood cells, and densities (parasites/μl of whole blood) were calculated with reference to the white cell count if available, or by assuming a count of 8 × 10 ^3^/μl if not. Haemoglobin types (HbA, HbS) were characterized by electrophoresis using cellulose acetate gels (Helena Laboratories, Beaumont, Texas, United States), while participants were typed for the common African 3.7-kb α-globin deletion by PCR as described previously [ 13, 14] to assign them thalassaemia genotypes. On the basis of previous work, we have found no evidence of β-thalassaemia in the population under study [ 15, 16]; further β-globin typing was not therefore conducted in these cohorts. Full blood counts were conducted using an automated cell counter (MDII; Beckman Coulter, Fullerton, California, United States) by standard methods.

### Statistical Analysis

We estimated the effects of categorical variables on disease outcomes through the calculation of odds ratios by both univariate and multivariate logistic regression. Continuous data were compared by linear regression. Variables that were not normally distributed were log-transformed prior to analysis. We estimated the effect of explanatory variables on the incidence of malaria and other diseases using Poisson regression. We used the Wald or likelihood ratio tests, as appropriate, to test for interactions between explanatory variables, and included interaction terms in our final models where significant evidence was found. Where a single individual contributed more than one data point, we took account of potential within-patient clustering of events using the sandwich estimator (as described by Armitage and colleagues [ 17]), which inflates confidence intervals and adjusts significance values as appropriate. All analyses were conducted using S TATA v 8.0 (Timberlake, London, United Kingdom).

Ethical permission for both studies was granted by the Kenya Medical Research Institute National Ethical Review Committee. Individual written informed consent was provided by all study participants or their parents.

## Results

In the mild disease cohort study, 4,296 clinic visits were made by 382 participants during 520.39 child years of follow-up (cyfu). Of these participants, 96 (25.1%) were of normal α ^+^-thalassaemia genotype (αα/αα), 149 (39%) were heterozygotes (−α/αα), and 56 (14.7%) were homozygotes (−α/−α). An additional 81 children (21.2%) participated in the study, but no samples were available for genotyping for them. These children were therefore excluded from the current analysis. Of the 301 participants with α ^+^-thalassaemia genotypes available, 39 (13%) were heterozygotes for Hb type (i.e., HbAS). The mean age (standard deviation [SD]) of participants during the period of study was 2.38 (1.50) y. The following seven diagnoses accounted for over 90% of consultations: malaria (by definition 1) (942/4,296 [27%]), upper respiratory tract infection (1,190 [28%]), lower respiratory infection (499 [12%]), gastroenteritis (490 [11%]), skin infection (478 [11%]), fever of unknown cause (135 [3%]), and helminth infection (199 [3%]). Consultations with other diagnoses and hospital admission events were too few to permit meaningful between-genotype comparisons.

In the birth cohort study, a total of 876 admissions were recorded among 2,104 participants during 8,181 cyfu. Of this cohort, 739 (35%) participants were of normal α ^+^-thalassaemia genotype, 1,017 (48%) were heterozygotes, and 348 (17%) were homozygotes. Of the 2,104 participants, 309 (15%) were Hb type heterozygotes, HbAS. The mean duration (SD) of follow-up of children contributing data to this analysis was 3.89 (1.02) y. Between them, malaria (434/876 [50%]), lower respiratory tract infections (236 [27%]), and gastroenteritis (68 [8%]) accounted for 85% of all admissions. Amongst the children admitted with malaria, 146/434 (34%) showed one or more signs of severity (as defined above). Of these episodes 60/146 (41%) could be defined according to one of two categories, cerebral malaria or severe malaria anaemia. The number of participants admitted with other diagnoses or malaria syndromes were too few to allow for meaningful comparisons.

### α ^+^-Thalassaemia and the Risk of Malaria

We found no evidence for any effect of α ^+^-thalassaemia on the prevalence of symptomless parasitaemia. During the four cross-sectional surveys combined, the prevalence was 59/320 (18.4%) in normal children, 100/543 (18.4%) in heterozygotes and 23/171 (13.5%) in those with homozygous α ^+^-thalassaemia, giving adjusted odds ratios for parasitaemia of 0.92 (95% CI, 0.62,1.38; *p* = 0.695) and 0.71 (0.37,1.39; *p* = 0.367) for heterozygotes and homozygotes, respectively, compared to normal children. Similarly, although the incidence of uncomplicated malaria (by either definition) was lower in both heterozygotes and homozygotes for α ^+^-thalassaemia than in normal participants involved in the mild disease cohort study, these differences were not significant either individually ( Table 1) or for both α ^+^-thalassaemia genotypes combined (incidence rate ratio [IRR] for definition 1, 0.83 [95% CI, 0.63,1.11; *p* = 0.206], and for definition 2, 0.90 [0.67,1.21; *p* = 0.471]). Nevertheless, α ^+^-thalassaemia was associated with significant reductions in the rate of admission to hospital with malaria and severe malaria measured through the birth cohort study. Homozygotes were admitted to hospital with malaria, with or without signs of severity, significantly less frequently than normal children ( Table 2). Similarly, heterozygotes were admitted significantly less frequently with severe malaria, cerebral malaria, and severe malaria anaemia. In the case of both genotypes, the lowest IRRs were for SMA. Although the incidence of cerebral malaria was lower in heterozygous than in normal children, of note, this result was true only when cerebral malaria was accompanied by anaemia: although numbers were small, we found no evidence for protection against cerebral malaria that was not complicated by anaemia. For comparison, the IRRs for each diagnosis by HbS category are shown in both Tables 1 and 2.

**Table 1:**
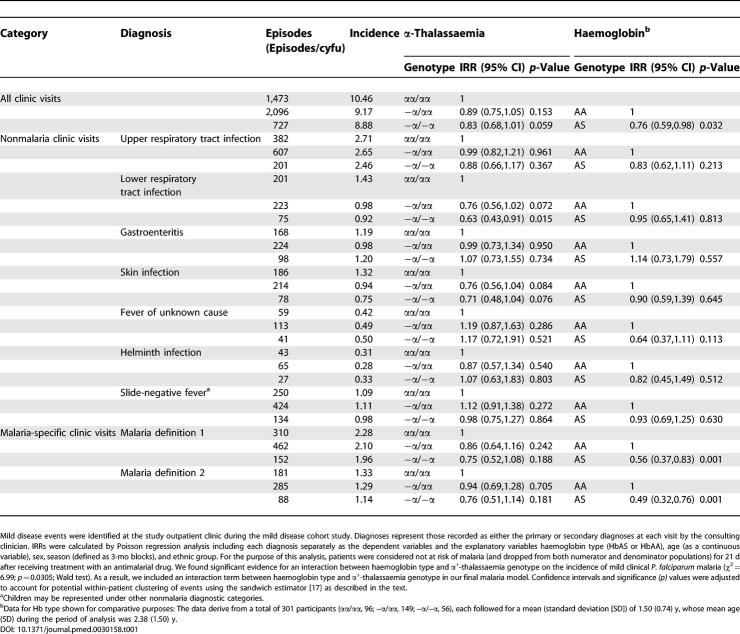
The Incidence of Mild Clinical Malaria and Other Diseases by α ^+^-Thalassaemia Genotype

**Table 2:**
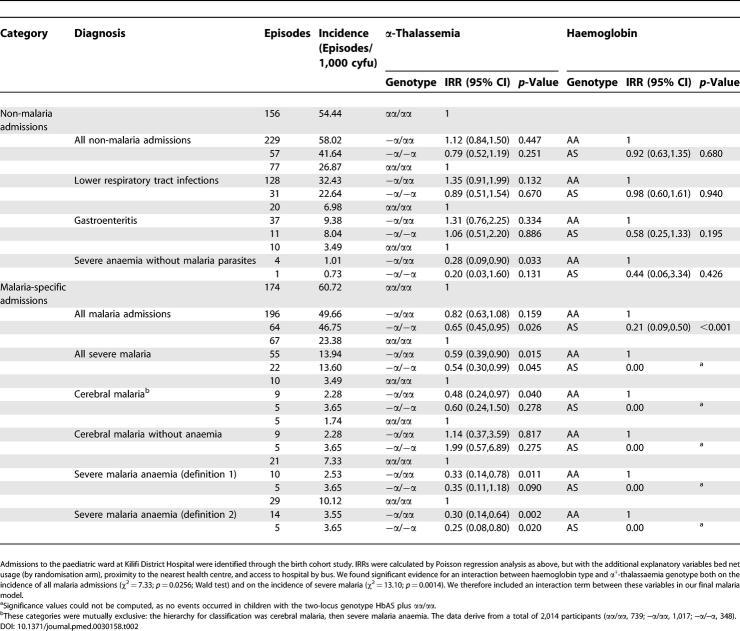
The Incidence of Severe Clinical Events by α ^+^-Thalassaemia Genotype

We found no significant associations between α ^+^-thalassaemia genotype and parasite density during incident infections, either when symptomless (detected through cross-sectional surveys) or when clinically apparent (detected through either the mild disease or birth cohort studies) ( Figure 1). This observation was also true after adjustments for age, season, and location.

**Figure 1:**
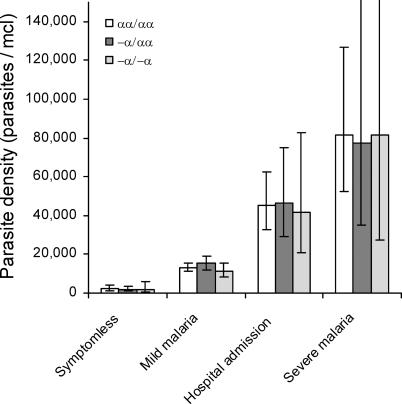
Parasite Densities by Clinical Status and α ^+^-Thalassaemia Genotype Geometric mean parasite densities are shown with 95% CIs. Data on symptomless parasitaemia reflect 59 measurements on normal (αα/αα) children, 100 on heterozygotes (−α/αα), and 23 on homozygotes (−α/−α) for α ^+^-thalassaemia. The equivalent figures for mild, hospital, and severe malaria are described in Tables 1 and 2. Between-genotype differences were tested using linear regression both with and without adjustments for age, season, and within-patient clustering. No significant differences were found.

### Nonmalaria Diseases

With only two exceptions, we found no significant association between α ^+^-thalassaemia genotype and the presence of clinical syndromes other than malaria ( Tables 1 and 2, Figure 2). First, in the birth cohort study, the incidence of severe anaemia leading to hospital admission was significantly lower in heterozygotes than in normal individuals (IRR 0.28 [95% CI, 0.09,0.90; *p* = 0.033]). Whereas in homozygotes, the numbers were too few to reach significance, the IRR for both genotypes combined was 0.26 (0.09,0.77; *p* = 0.015). Second, the incidence of lower respiratory tract infections, detected during the mild disease cohort, was significantly lower in both heterozygous and homozygous than in normal children ( Table 1); however, the same was not true for lower respiratory infections detected through the birth cohort study ( Table 2). IRRs by HbS phenotype for each diagnosis are shown for comparison. An analysis of the incidence rates for both malaria and nonmalaria diseases in the group of 81 study participants without α ^+^-thalassaemia genotypes did not suggest that their exclusion introduced any significant bias.

**Figure 2:**
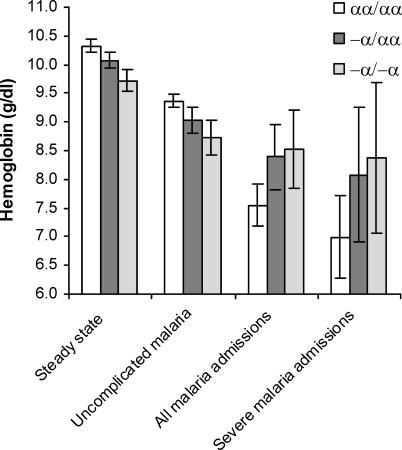
Haemoglobin Concentrations at Steady State and During Episodes of Clinical P. falciparum Malaria Values are means (with standard errors). 1 g/dl = 10 g/l Data for steady state, all malaria admissions, and severe malaria admissions derive from the birth cohort study. Severe malaria was defined as described in the text. Study participants (and genotypes) numbered as follows: steady state *n* = 2,104 (αα/αα, 739; −α/αα, 1,017; −α/−α, 348); all malaria admissions *n* = 434 (αα/αα, 174; −α/αα, 196; −α/−α, 64); and severe malaria admissions *n* = 146 (αα/αα, 67; −α/αα, 55; −α/−α, 24). Data for uncomplicated malaria derive from the mild disease cohort study. Data for αα/αα reflect 420 measurements in 96 study participants; for −α/αα are from 701 in 149; and for −α/−α are from 212 in 56. Amongst children in steady state, mean difference (β) = −2.6 g/l (95% CI, −4.1,−1.1; *p* = 0.001) and −5.6 (−7.8,− 3.8; *p* < 0.001) for −α/αα and −α/−α, respectively. The equivalent β values for uncomplicated malaria were −3.2 (−5.6,−7.7; *p* = 0.010) and −6.8 (−10,−3.4; *p* < 0.001); for hospital-admitted malaria were 8.4 (2.8,14.1; *p* = 0.003) and 0.97 (0.29,1.65; *p* = 0.005); and for severe malaria were 1.08 (−0.10,2.25; *p* = 0.072) and 13.8 (0.60,26.9; *p* = 0.041). These figures were adjusted for age (as continuous) and sex, and for potential within-person clustering where children contributed more than one data point.

### Haematological Indices

Haemoglobin concentration was significantly lower in both heterozygotes and homozygotes for α ^+^-thalassaemia both at steady state and during clinical episodes of P. falciparum malaria ( Figure 2). However, this association was reversed during episodes of more severe malaria presenting to hospital, both when uncomplicated and when complicated by any of the standard criteria we use to define severe malaria ( Figure 2).

## Discussion

In two large cohort studies conducted on the coast of Kenya, we found no associations between α ^+^-thalassaemia and the prevalence of symptomless P. falciparum parasitaemia, the incidence of uncomplicated P. falciparum disease, or parasite densities during mild or severe malaria episodes. However, we found significant negative associations between α ^+^-thalassaemia and the incidence rates of severe malaria and severe anaemia, the strongest associations being for severe malaria anaemia and severe nonmalaria anaemia.

The evidence for malaria protection by the α ^+^-thalassaemias is now overwhelming [ 14, 18– 22]. Because of this protection, these genes have been selected to population frequencies that are higher than those of any other human genetic polymorphism described to date, yet the mechanisms of protection are poorly understood. Although several mechanisms are supported by studies conducted in vitro (summarized in [ 23]) it is difficult to gauge their relevance in the absence of clear descriptions of the effects of α ^+^-thalassaemia in populations naturally exposed to malaria. To date, such descriptions have proved confusing. For example, although α ^+^-thalassaemia confers protection against both severe [ 14, 21, 22] and fatal [ 14] malaria, until now the evidence has tended to suggest a raised, rather than a reduced, incidence of uncomplicated malaria in such children [ 24, 25]. Furthermore, data from a case-control study conducted in Papua New Guinea suggested that the selective advantage of α ^+^-thalassaemia may not be confined to malaria, but may also extend to other diseases [ 21].

We aimed to clarify some of these issues through epidemiological studies of both malaria and non-malaria diseases conducted in a malaria-endemic area on the coast of Kenya. As expected, we found significant evidence for protection against the more severe forms of P. falciparum malaria that result in hospital admission. In keeping with data from recent case control studies [ 14, 22], these effects were most marked for the most severe forms, particularly SMA. Moreover, in keeping with previous studies [ 26, 27], we found no effect of α ^+^-thalassaemia either on parasite density or on the prevalence of symptomless parasite carriage. We did see some evidence towards a reduced incidence of uncomplicated malaria in α ^+^-thalassaemic children ( Table 1), although this result was not significant, either in children of each genotype individually or in both genotypes combined. This observation contrasts with that from an earlier study, conducted on the Pacific islands of Vanuatu, in which we found that the incidence of uncomplicated malaria was paradoxically raised in children with both heterozygous and homozygous α ^+^-thalassaemia [ 25]. Those findings led us to speculate that a predisposition to mild clinical malaria might result in the accelerated acquisition of protective immunity through a natural form of vaccination. Although our current study does not appear to support this hypothesis, it does not exclude it for several reasons. For example, there are marked differences in the epidemiology of malaria between the two study areas. Unlike Kilifi, where most malaria episodes are caused by P. falciparum, P. vivax accounted for roughly half the episodes in Vanuatu [ 25, 28, 29]. Similarly, the relative incidence of malaria caused by the two dominant species varied with age: P. vivax predominated in the early years of life and was not superseded by P. falciparum until after the age of 2 y, an observation suggesting that there might be a biological interaction between the two species [ 30]. Moreover, there are striking differences between the two areas in terms of the genetic background of both the human and the parasite populations [ 31, 32]. Finally, our current study of mild clinical disease included relatively few children in the youngest age classes, and it is therefore possible that it lacked statistical power to show a significant interaction between malaria protection and age. Further studies will be required to dissect this important question.

With only two exceptions, we found no significant associations between α ^+^-thalassaemia and nonmalaria disease. First, the incidence of lower respiratory tract infections, diagnosed in the outpatient clinic during the mild disease cohort study, was significantly lower in homozygotes than in normal children ( Table 1). There was no significant difference in the incidence of lower respiratory infections in heterozygotes overall, nor did we find any associations with the incidence of more severe lower respiratory tract infections that resulted in hospital admission ( Table 2). While it seems possible, therefore, that the observation regarding respiratory infections in the mild disease cohort represents a chance finding, it remains plausible that a true effect exists for two reasons: (1) The observation was based on a large number of episodes, and the strength of the effect was of the same order as that observed for malaria in the same cohort; and (2) because in the birth cohort information was available only regarding the disease experience of children who survived to 5 to 8 y of age (the age at which blood samples were collected for genotyping), it is possible that the lack of effect of α ^+^-thalassaemia on the incidence of respiratory tract infections in this cohort might be biased by our study design. These observations are pertinent in light of the protective association against nonmalaria hospital admissions that we found in Papua New Guinea [ 21]. That finding seemed plausible for a number of reasons: it could have been mediated through the prevention of acute malaria episodes, which may be accompanied by some degree of immunosuppression [ 33], or possibly through the improved acquisition of nonspecific, antidisease immunity to malaria that might also protect against the consequences of other infections [ 34]. A further possibility is that it could have resulted from some degree of misclassification, as in most tropical settings it is difficult to differentiate common causes of childhood illness on the basis of clinical criteria alone. Our current study is inconclusive with regard to respiratory tract infections: Further work addressing specific syndromes, such as pneumonia or invasive bacterial disease, will be required to address these issues definitively.

The second significant observation regarding α ^+^-thalassaemia and nonmalaria disease was the reduced incidence of severe nonmalaria anaemia in children admitted to hospital. In light of the reduced incidence of SMA, we speculated that even in the absence of detectable parasites at the time of admission, the aetiology of this anaemia might also relate to malaria. With a view to investigating these relationships further, we stratified haemoglobin concentration by α ^+^-thalassaemia genotype, both in study participants at steady state and in those affected by clinical episodes of P. falciparum malaria. In keeping with previous reports [ 27, 35– 37], we found a significant negative correlation between α ^+^-thalassaemia and haemoglobin concentration at steady state ( Figure 2). While this correlation held up in children presenting with mild clinical malaria, it was reversed in children presenting with the more severe forms, either resulting in admission to hospital or classifiable as severe according to standard criteria ( Figure 2). This finding suggests the possibility that α ^+^-thalassaemia may protect against the progression of anaemia during the course of clinical episodes of P. falciparum disease.

Anaemia is a common sequel of malaria infections, but is often clinically silent [ 38, 39]. As such, it likely makes a large but hidden contribution to overall malaria mortality, especially in young children [ 38]. Mortality is greatest when anaemia is severe (Hb < 50 g/l), and complicated by other signs of severity [ 40]. Two factors correlate best with the development of severe anaemia: haemoglobin concentration preceding the malaria transmission season, and the parasite density achieved during incident infections [ 41]. We found no evidence for an effect of α ^+^-thalassaemia on either of these parameters. Consistent with observations in other malaria-endemic populations, we found a negative correlation between α ^+^-thalassaemia genotype and haemoglobin concentration at steady state [ 35, 36, 42]. Similarly, in keeping with previous studies [ 14, 21, 22, 26, 27, 42, 43], we found no effect of α ^+^-thalassaemia on parasite densities during malaria infections. Moreover, it seems unlikely that anaemia protection was mediated by a favourable iron status in α ^+^-thalassaemic children, as we found no correlation between α ^+^-thalassaemia genotype and biochemical markers of iron status in a recent study conducted in the same area [ 44]. We suggest, therefore, that our observations reflect protection against the progression of individual malaria infections to the point at which they result in severe anaemia.

Severe malaria anaemia is probably mediated by a number of processes that may include both acute haemolysis and suppression of normal eythropoiesis [ 38, 45]. It is not clear how α ^+^-thalassaemia might prevent progression to SMA; however, recent observations by Cockburn and colleagues [ 46] regarding the expression of complement receptor 1 (CR1) on the surface of α ^+^-thalassaemic red blood cells might prove relevant. In an extension of a previous case control study conducted in Papua New Guinea [ 21], these workers made two related observations. First, they found a protective association between a promoter polymorphism of red cell CR1 and severe falciparum malaria, and second, they found an independent negative association between the expression of red cell CR1 and α ^+^-thalassaemia genotype. Previous work suggests that CR1 is an important receptor for rosetting, a phenomenon whereby uninfected red blood cells bind to *P. falciparum*-infected erythrocytes in vitro and that is implicated in the pathogenesis of severe and complicated disease [ 47– 50]. These observations therefore provide potential mechanism for both the protective effect of α ^+^-thalassaemia and the earlier observation that α ^+^-thalassaemic red cells are less able to form rosettes in vitro [ 51, 52]. The enhanced expression of antigens on the surface of late-stage P. falciparum-infected red blood cells that has been identified in a number of studies [ 53, 54], may provide an alternative mechanism—by resulting in their early or enhanced removal from circulation. However, we would anticipate that if this were the mechanism, it would be reflected in reduced parasite densities as are seen in participants with an HbAS genotype [ 12]. Further studies investigating the relationships between α ^+^-thalassaemia, red cell CR1 expression, rosetting, immune clearance, and the various clinical phenotypes of severe malaria may therefore be informative regarding the pathophysiological processes involved.

The implications of our study for the interpretation of malaria intervention studies, particularly those based on vaccines, and for studies that aim to identify new malaria-protective genes, are worthy of note. It is clear from both our current ( Table 2) and previous studies [ 14, 21, 22], that α ^+^-thalassaemia is strongly protective against severe and complicated malaria, but has no effect at all on either the prevalence of symptomless parasitaemia or on parasite densities during incident malaria infections. Furthermore, despite an apparently well-powered study capable of demonstrating a marked effect of HbAS, we found no significant effect of α ^+^-thalassaemia on the incidence of mild, uncomplicated malaria disease events ( Table 1). These observations argue for careful consideration of which disease phenotype to measure when designing studies of this sort. They suggest that wherever possible, well-characterized phenotypes of severe and complicated disease or, even better, malaria-specific mortality, are the outcomes of choice.

**Patient Summary**

**Background**

Malaria is a very common disease in African children, although a number of factors influence how severe the disease is, including the age and genetic background of the child. Changes in the genes that code for the globin proteins (alpha and beta) that are an essential part of hemoglobin (the oxygen-carrying protein in the blood) are known to influence how people are affected by malaria. For example, one such change in the beta globin gene causes sickle hemoglobin; when individuals have two copies of the affected gene they can be severely affected with anemia and painful crises, but carrying just one copy seems to make people less susceptible to malaria. Other genetic variants, in the alpha globin gene, cause a condition called alpha-thalassaemia, which can also lead to anemia because of lower amounts of the alpha globin protein being present. People with alpha thalassaemia seem to have some protection against malaria.

**Why Was This Study Done?**

Both sickle cell and alpha-thalassaemia are very common in Africa. These investigators wanted to investigate whether having alpha-thalassaemia protects against all types of malaria or just the severe type.

**What Did the Researchers Do and Find?**

The authors studied two groups of children living on the coast of Kenya to find out whether they had alpha-thalassaemia. They assessed whether or not having alpha-thalassaemia led to any difference in whether the children were infected with the parasite that causes malaria, whether they had symptoms, and how severe the symptoms were. They found that having alpha-thalassaemia meant that the children were less likely to get severe malaria and severe malarial anemia. However, there was no effect on whether the children got mild malaria or not.

**What Do These Findings Mean?**

These results suggest that over generations the genetic change that causes alpha-thalassaemia has become common in this population, because carrying it protects people against getting severe malaria, especially with anemia. However, the genetic change does not appear to make it more or less likely that children get mild malaria. This result may be important when designing studies to look at the relation between these genetic changes and malaria.

**Where Can I Get More Information Online?**

Medline Plus has a page of links to malaria:

http://www.nlm.nih.gov/medlineplus/malaria.html

and a page discussing thalassaemia:

http://www.nlm.nih.gov/medlineplus/ency/article/000587.htm

## Abbreviations

CI: confidence interval
CR1: complement receptor 1
cyfu: child years of follow-up
Hb: haemoglobin
IRR: incidence rate ratio
SD: standard deviation
SMA: severe malaria anaemia
